# Interactions Between Microplastics and Heavy Metals in Aquatic Environments: A Review

**DOI:** 10.3389/fmicb.2021.652520

**Published:** 2021-04-22

**Authors:** Sitong Liu, Jiafu Shi, Jiao Wang, Yexin Dai, Hongyu Li, Jiayao Li, Xianhua Liu, Xiaochen Chen, Zhiyun Wang, Pingping Zhang

**Affiliations:** ^1^School of Environmental Science and Engineering, Tianjin University, Tianjin, China; ^2^Fujian Provincial Engineering Research Center of Rural Waste Recycling Technology, College of Environment and Resources, Fuzhou University, Fuzhou, China; ^3^College of Food Science and Engineering, Tianjin Agricultural University, Tianjin, China

**Keywords:** microplastics, heavy metals, interactions, biofilm, microbe, aquatic environment

## Abstract

Microplastics (MPs), tiny particles broken down from larger pieces of plastics, have accumulated everywhere on the earth. As an inert carbon stream in aquatic environment, they have been reported as carriers for heavy metals and exhibit diverse interactive effects. However, these interactions are still poorly understood, especially mechanisms driving these interactions and how they pose risks on living organisms. In this mini review, a bibliometric analysis in this field was conducted and then the mechanisms driving these interactions were examined, especially emphasizing the important roles of microorganisms on the interactions. Their combined toxic effects and the potential hazards to human health were also discussed. Finally, the future research directions in this field were suggested. This review summarized the recent research progress in this field and highlighted the essential roles of the microbes on the interactions between MPs and heavy metals with the hope to promote more studies to unveil action mechanisms and reduce/eliminate the risks associated with MP presence.

## Introduction

Microplastics (MPs) have received global attention due to their ubiquitous presence in the environment and unknown hazards to organisms. Compared with the traditional plastic waste, the distinct character of MPs is their small size, which is precisely defined as plastic fragments and particles with a diameter of less than 5 mm (Thompson et al., 2004). There are two types of MPs: primary and secondary. Primary MPs may be intensively produced for various purposes, such as microbeads in commercial facial cleansers and resin particles (Bayo et al., 2017; So et al., 2018). Secondary MPs are decomposed from large pieces of plastic waste during environmental processes such as aging, weathering, and biodegradation (Gouin et al., 2011).

MPs can be accumulated in the environment due to their inert nature (Wang et al., 2021a). They have been proven to be widely distributed in our environment. For example, the abundance of MPs in the sediments of tropical Atlantic Ocean is 684.8 particles/m^2^, and most of them are round particles and fragments with different shapes (Benson and Fred-Ahmadu, 2020). MPs have also been found in the sediments of China’s three largest lakes (Zhang B. et al., 2020). Alarmingly, in Qinghai-Tibet Plateau, which has low plastic consumption and population density, high abundance of MPs was detected, which illustrates the wide distribution of MPs. In addition, as an emerging complex pollutant, MPs can produce various toxic effects on organisms. The exposure of aquatic organisms to MPs has been associated with short- and long-term adverse effects on organism’s health, including biological feeding, reproduction, antioxidant defense and innate immunity (Murphy and Quinn, 2018; Wen et al., 2018a; Banaee et al., 2019; Oliviero et al., 2019).

Heavy metals are present in the environment from both naturally occurring and anthropogenic sources. As a contaminant that is widespread in the environment, heavy metals can enter water bodies continuously due to their non-degradable nature and are recycled and enriched in the aqueous environment. MPs and heavy metals are not only acting as persistent pollutants, their combined pollution poses a new threat to the world. Due to the large surface area, MPs can act like magnets for toxic pollutants and concentrate them to a very high level. Heavy metals had been found on the MPs from North Atlantic subtropical gyre (Prunier et al., 2019), São Paulo State in southeastern Brazil (Vedolin et al., 2018), beaches in southwest England (Massos and Turner, 2017), and western Europe (Turner et al., 2019).

Aquatic ecosystems contain a great diversity of microorganisms, which play critical roles in many biogeochemical processes. Their existence makes the interaction between MPs and heavy metals more complicated. Firstly, MPs can provide emergent ecological niche for microorganisms by formation of microbial biofilms, named plastisphere (Mincer et al., 2016; Yang et al., 2020). In the plastisphere, MPs can offer firm support for microbial colonization and even be served as carbon sources for microbial growth. Studies have shown that MP-associated biofilms could affect the physical and chemical properties of MPs and further affect the adsorption of chemical pollutants including heavy metals (Tu et al., 2020). Secondly, exposure to heavy metals can impact biofilm formation and bring structural changes to the biofilm matrix, which further impact the adsorption behavior of heavy metals (de Araújo and de Oliveira, 2020). Although the role played by biofilms has become a hot spot in the MP studies, their influence on the fate of MPs and heavy metals is still not well understood.

As a whole, a great deal of work has been done to analyze the type, size, shape, color, and abundance of MPs in order to explain the source, sink, and destination of MPs in the environment. In the meantime, many types of heavy metals have also been detected on the surface of MPs, which showed that MPs can be unneglectable carriers of heavy metals. As a crucial biotic components in aquatic systems, microbes may also play a key role in modulating the interaction between MPs and heavy metals. Until now, there is still lack of comprehensive review papers on the interactions between MPs and heavy metals. This review aims to fill in this gap and highlight key future research areas in this field. The microbe’s important roles on the interactions will also be emphasized. The following aspects will be focused on (1) conducting a bibliometric analysis in this field to summarize the recent research progress and trend, (2) examining the interaction behaviors and underlying mechanisms between MPs and heavy metals, (3) discussing the combined toxic effects and the potential hazards to human health, and (4) suggesting the future research directions in this field. This review will help to deepen the understanding of the sources, transport routes, and ecological risks of MPs and heavy metals, and promote the actions to reduce their possible risks on the ecological system and human health.

## Bibliometric Analysis

In order to better understand the current state of research in the field of MPs and heavy metals, a bibliometric analysis was conducted. The original literature data were collected from the Web of Science with the search strategy of “microplastic^∗^ and (‘heavy metal^∗^’ or copper or lead or zinc or iron or cobalt or nickel or manganese or cadmium or mercury),” and a total of 978 papers published between 2006 and 2021 were identified. The software CiteSpace 5.7.R3 was used to conduct burst keywords analysis and co-occurrence keyword network analysis, and Gephi 0.9.2 was employed to visualize the keywords network.

**FIGURE 1:**
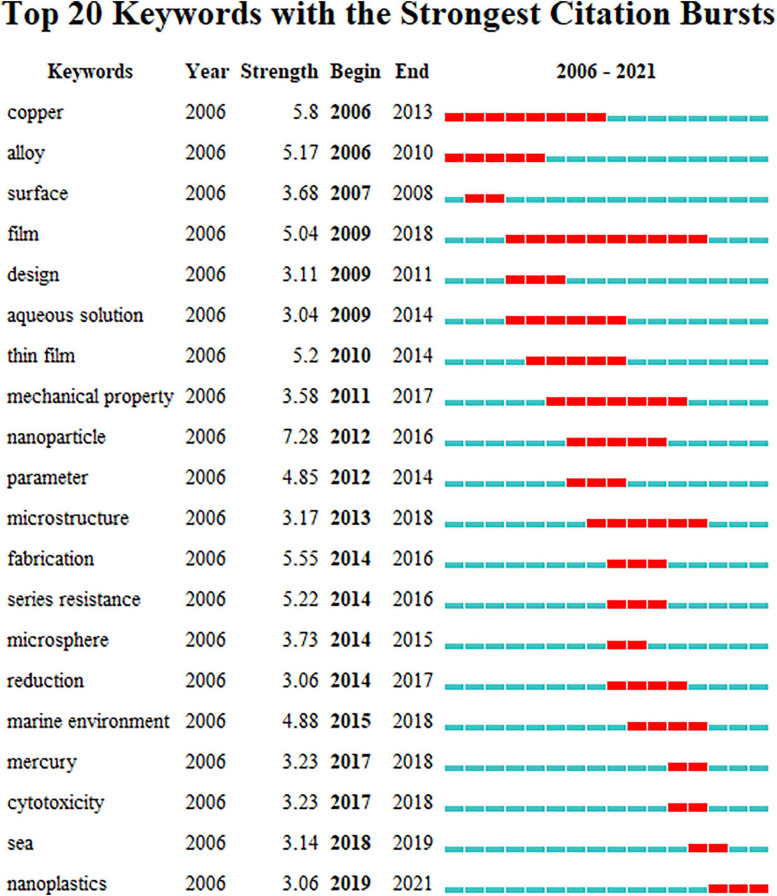
Top 20 keywords with the strongest citation bursts during 2006–2021.

**FIGURE 2:**
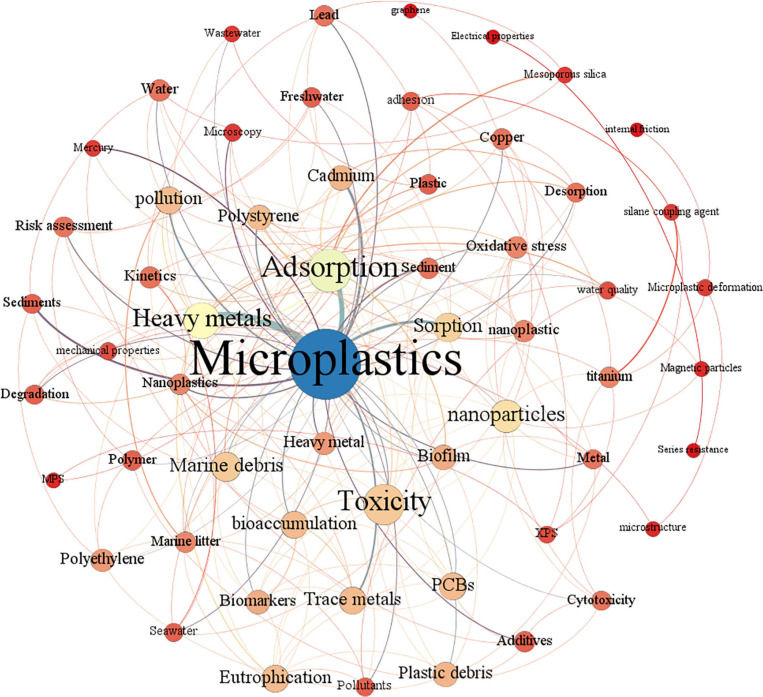
Co-occurrence analysis of keywords with a word frequency of more than five times. The data come from a literature search with MPs and heavy metals as the subject terms. Each node is represented as a keyword, edges represent the link between keywords, the color of the node is rendered in degrees, and the color of the edge is rendered in the weight of the edge.

**FIGURE 3:**
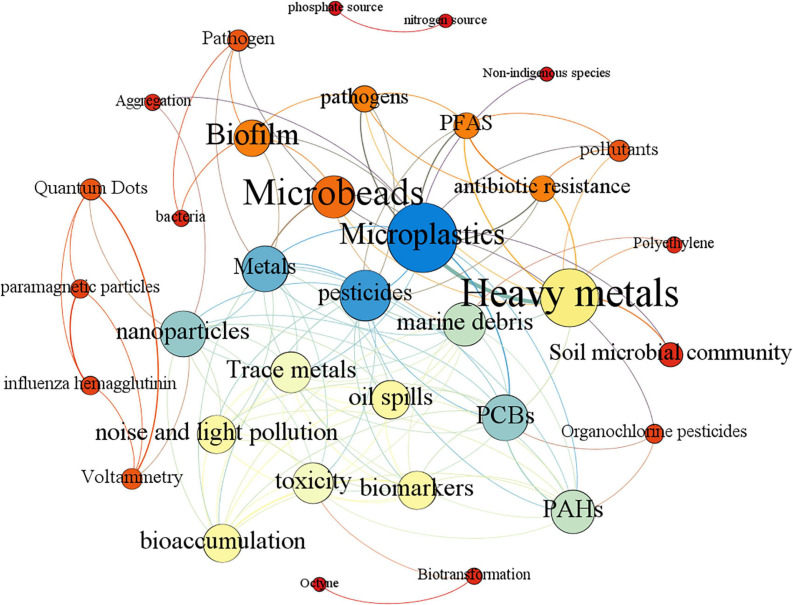
Co-occurrence analysis of keywords with a word frequency of more than five times. The data come from a literature search with MPs, heavy metals, and microbes as the subject terms. Each node is represented as a keyword, edges represent the link between keywords, the color of the node is rendered in degrees, and the color of the edge is rendered in the weight of the edge.

Burst keywords can reflect emerging trends and hot spots. Figure 1 shows the burst keywords detection result between 2006 and 2021. Among all the burst keywords, the keyword nanoparticle has the strongest burst intensity (7.28), which is much higher than other keywords, indicating that research related to MPs is no longer limited to the micron level, but has a tendency to move to the nanometer level, which is also confirmed by the keyword nanoplastics becoming an emerging buzzword in 2019–2021. As one of the most popular heavy metals related to MP study, copper has the second burst intensity (5.8). Among all the popular keywords, the keyword film has the longest duration of hotness, receiving strong attention for 10 consecutive years from 2009 to 2018, which indicates that there is still a large amount of research focusing on basic research in the field of MPs. The rise of the keyword cytotoxicity means that the research on the toxic effects of MPs and heavy metals at the cellular level is being further developed. Keywords with frequencies over five times were analyzed by co-occurrence mapping analysis (Figure 2). MPs, heavy metals, and adsorption were the top three keywords with the highest frequency and degree and were closely linked to other nodes. At the same time, in order to better understand the role of microbes in this field, keyword co-occurrence mapping analysis on MPs, heavy metals, and microbes was also conducted (Figure 3). Among the top keywords, biofilm has a high betweenness centrality and degree, demonstrating that it plays an important role in the interaction network between heavy metals and MPs. However, there are not many literature reports on topic, and only 94 papers were retrieved, implying that this field still has a great potential for further development.

## Interaction Characteristics and Driving Mechanisms

### Influence of Environmental Factors on Interactions Between MPs and Heavy Metals

Table 1 lists impacts of different environmental factors on the interactions between MPs and heavy metals. These factors include aging, temperature, pH, contact time, ionic strength, and particle size. Aging, whether with UV irradiation or with aging agents such as H_2_O_2_ and Fenton, can increase the adsorption capacity of MPs for heavy metals (Wang et al., 2019a). This observation was consistent across different studies (Mao et al., 2020; Lang et al., 2020; Wang Q. et al., 2020). The mechanism for enhanced adsorption may be due to the increase of specific surface area and oxygen-containing functional groups after aging. Compared with the unaged PET, new ketone groups were found on the surface of the PET aged by UV (Wang Q. et al., 2020). Photodegradation can also break the bonds on the MP surface and form new carboxyl groups (Bandow et al., 2017; Liu et al., 2019). These oxygen-containing groups can increase the polarity of MPs (Holmes et al., 2012) and make the MP surface more reactive (Holmes et al., 2014), leading to an increase of the adsorption capacity for metal ions. In terms of pH, generally only the case of pH < 7 will be considered because metal ions will precipitate under alkaline conditions. The pH can significantly affect the adsorption capacity of MPs to heavy metals. When pH is less than a certain value, MPs will not interact with heavy metals (Tang et al., 2020). Generally, increased pH level results in increased adsorption capacity for heavy metals (Guo et al., 2020); however, there are also different opinions (Zhang W. et al., 2020). The different observations may be due to the use of different heavy metals in these studies. Metal ions in the former case were usually positively charged, such as Cd^2+^, while in the latter case, they were normally negatively charged, such as CrO_4_^2–^. Taking the latter as an example, when the pH is less than 3, the surface of the MPs (PE) is positively charged, and the zeta potential and electrostatic repulsion are low. The negatively charged CrO_4_^2–^ are more likely to be strongly attracted by the positively charged MPs. However, with the increase of pH, the surface of the MPs (PE) becomes negatively charged and the adsorption capacity for CrO_4_^2–^ decreases. As for the temperature, the general opinion is that high temperature will benefit the adsorption of heavy metals on MPs (Oz et al., 2019; Wang T. et al., 2020). The possible explanation for this observation is that the adsorption process is an endothermic reaction; thus, the spontaneity of the adsorption process may increase with the increase of temperature.

**TABLE 1:** Research on the characteristics of interaction between MPs and heavy metals.

**Types of MPs**	**Types of heavy metals**	**Factors**	**Results**	**References**
PE PP PS PVC	Cd^2+^	pH	The sorption tendency increased as the pH increased, PVC > PS > PP > PE	Guo et al., 2020
		Ionic strength	The sorption capacity decreased as the salinity increased, PVC > PS > PP > PE	
		Humic acid (HA)	The sorption tendency increased as the HA increased, PVC > PS > PP > PE	
PA	Pb^2+^	pH	The minimum adsorption efficiency (%) was 3.37% at pH 2.5, the maximum adsorption efficiency (%) of 91.24% was acquired at pH 6.	Tang et al., 2020
PE	Cr^3+^	pH	The adsorption capability was increased when the dosage of PE MPs was increased	Zhang W. et al., 2020
		SDBS	The addition of SDBS can improve the adsorption capacity of PE on Cr^3+^. The peak of the adsorption capacity was at SDBS concentration between 1 and 1.5 mM	
		SDBS and pH	pH < 6, with the increase of SDBS, the adsorption efficiency increases. pH > 6, SDBS would compete with CrO_4_^2+^ for occupying the adsorption sites of PE microplastic.	
PET PA EVA	Pb^2+^	pH	pH is the most significant factor, the maximum adsorption was acquired at pH 5.5.	Oz et al., 2019
		Temperature	Adsorption capacity increases for PET, PA, and EVA with increasing temperatures but it was not affected much.	
PE	Cr	Concentration of chromium	The higher the initial concentration of chromium, the higher the adsorption capacity	Zon et al., 2018
PET	Zn^2+^ Cu^2+^	Aging	There is a positive correlation between the degree of aging and the adsorption capacity	Wang Q. et al., 2020
		Microplastic dosage	The more MP doses, the higher sorption capacity of metal ions was fully realized	
		Time	The longer the adsorption time, the greater the adsorption capacity.	
		pH	The pH range is 3–7; the higher the pH, the greater the adsorption capacity.	
		Temperature	The temperature range is 288K–318K; the higher the temperature, the greater the adsorption capacity.	

*PA, Polyamide; PE, Polyethylene; PET, Polyethylene Terephthalate; PP, Polypropylene; PS, Polystyrene; PVC, Polyvinyl Chloride; EVA, Ethylene Vinyl Acetate Copolymer; SDBS, Sodium Dodecyl Benzene Sulfonate.*

### Adsorption Kinetics and Isotherms of Heavy Metals on MPs

The kinetic study of the adsorption process can describe the rate of heavy metal adsorption by MPs, and the fitting of the kinetic model allows further analysis of the adsorption mechanism. Commonly used adsorption models include pseudo-first-order kinetic model, pseudo-second-order kinetic model, Elovich kinetic model, Boyd model, Weber–Morris Model, and Bangham channel diffusion model. The adsorption process was usually fitted with a pseudo first-order kinetic model (Zhang et al., 2018; Zon et al., 2018); however, some researchers also reported that the pseudo-second-level kinetic model can yield a better fit (Nethaji et al., 2013; Taha et al., 2016; Oz et al., 2019; Guo et al., 2020; Tang et al., 2020). This model assumes that the adsorption of heavy metals by MPs is mainly controlled by the chemisorption mechanism, involving the sharing or transfer of electron pairs, and is not controlled by the material transport step. The Weber–Morris model was used to describe the multistage nature of the adsorption process (Nethaji et al., 2013; Taha et al., 2016; Zon et al., 2018; Oz et al., 2019). In this model, adsorption may occur in multiple steps. Guo et al. (2015, 2020) divided the adsorption process into three steps. The first step is the rapid combination of heavy metal ions with active sites on MP surface, which is mainly attributed to the covalent and van der Waals forces. When this binding process reaches a saturation, due to the increased diffusion resistance, the adsorption enters the second step, and the heavy metal begins to diffuse slowly into the pores of the MP particles. In the final step, the adsorption rate decreases significantly, eventually reaching an equilibrium state between adsorption and desorption.

The adsorption isotherm can be used to describe the distribution of pollutants between the solid and liquid phases in the adsorption equilibrium state, and the most commonly used models are the Langmuir isotherm model (Langmuir, 1918) and the Freundlich isotherm model. The Langmuir isotherm model assumes that there is no interaction force between the adsorbed molecules and only monolayer adsorption can be formed on the MP surface, while the Freundlich isotherm model is an empirical equation with no assumptions. Some researchers reported that the adsorption process of MPs for heavy metals can be described by the Langmuir isotherm model (Tang et al., 2020; Wang T. et al., 2020; Zhang S. et al., 2020), while some others declared that the Freundlich isotherm model was better (Fang et al., 2019; Purwiyanto et al., 2020; Shan et al., 2020). In the Freundlich isotherm model, the adsorption process is a multilayer adsorption that occurs on a heterogeneous surface, and pollutant molecules will first occupy high-energy adsorption sites and then diffuse to low-energy adsorption sites (Abdurahman et al., 2020; Wang T. et al., 2020). There are also studies showing that both Langmuir and Freundlich models can successfully describe adsorption isotherms (Zon et al., 2018; Dong et al., 2020).

In general, the adsorption mechanism between MPs and heavy metals can be generally described by electrostatic interactions, van der Waals forces, and π–π interactions (Fu et al., 2020; Lin et al., 2021; Torres et al., 2021). It was shown that all MPs have a pH point of zero charge (pHpzc) around 3, which implies that in the natural aqueous environment, MPs should carry a negative charge on their surface (Lin et al., 2021). The electrostatic attraction between these negatively charged MPs and positively charged metal ions promotes the adsorption behavior. Compared to electrostatic interactions, van der Waals forces and π–π interactions play a relatively small role. However, they can further promote the sorption capacity (Torres et al., 2021). Their occurrence depends on the polymer type; e.g., PE (aliphatic polymer) exhibits van der Waals interactions, while PS (aromatic polymer) exhibits mainly π–π interactions. Different polymers have different polarity, crystallinity, and surface functional groups, resulting in different adsorption behavior during adsorption (Kim et al., 2017; Chen et al., 2019; Loncarski et al., 2020). Guo et al. (2020) studied the adsorption of cadmium ions by four types of MPs (PP, PE, PS, and PVC) and found that the order of adsorption capacity was PVC > PS > PP > PE. Llorca et al. (2020) also reported that PET and PS had higher affinity for pollutants than PE. As simple non-polar crystalline polymers, PE and PP have no functional groups and can only adsorb contaminants in a single layer with van der Waals forces, so the adsorption capacity is relatively small (Chen et al., 2019). For PS and PET, the polarity is increased due to the presence of phenyl and ester groups, and the adsorption capacity can be increased through the interaction of π–π bonds with pollutants (Llorca et al., 2020; Loncarski et al., 2020). PVC, because it contains polar atomic chlorine, is a strong polar polymer, so it has very large adsorption capacity (Brennecke et al., 2016).

### Role of Microbes on the Interactions Between MPs and Heavy Metals

The aquatic environment is a highly complex ecosystem, and there are dynamic interactions between biotic and abiotic components (de Araújo and de Oliveira, 2020). In addition to physical and chemical environmental factors, biological factors can affect the adsorption of heavy metals by MPs. The influence of microbial biofilm is one of the most concerned biological factors and it plays an important role in determining the MP surface properties. Tu et al. (2020) found that biofilm formation reduced the hydrophobicity of the PE MP surface and increased the abundance of carboxyl and ketone groups on the MP surface, which then increased the adsorption capacity for metal ions. Some studies revealed that the growth of biofilms can positively affect the adsorption of heavy metals and concentration of heavy metals on MPs will increase as the biofilm matures (Richard et al., 2019; Qi et al., 2021). It must be noted, however, that the long-term dynamic change of heavy metal loads on MPs during the whole biofilm development remains largely unknown. Sinking of floating MPs caused by biofilm development further complicates the problem (Rummel et al., 2017). Much more work need to be done to quantitatively determine the influence of biofilm on heavy metal adsorption for a better understanding of their interactions in the aquatic environment. In addition, Jin et al. (2020) reported how biofilm on MPs affects the uptake and fate of hydrophobic organic compounds. They found that the high temperature in the summer was more favorable for the colonization of highly active bacteria on the MP surface, and interactions between pollutants and attached microorganisms in the biofilm essentially depend on pollutant features and microbial activity.

**FIGURE 4:**
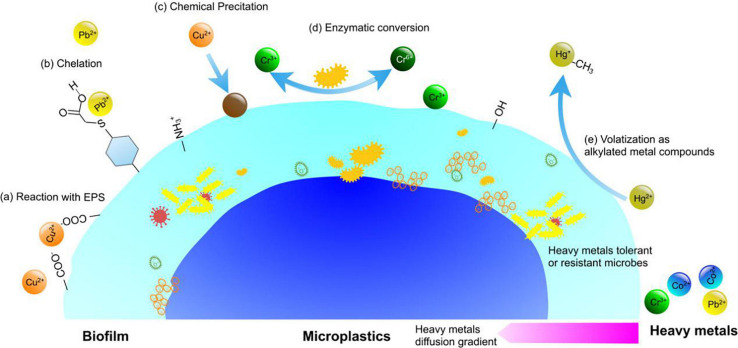
Mechanisms of biofilm involved in the interactions between MPs and heavy metals. **(a)** Reaction with extracellular polymeric substance (EPS) in the matrix; **(b)** chelation with proteins and peptides; **(c)** precipitation via chemical or biological agents; **(d)** enzymatic conversion; **(e)** volatilization as alkylated metal compounds.

In the meantime, environmental factors may indirectly exert influence on MPs by changing biofilm structures on the MP surface. Wang L. et al. (2020) found that nutrient salts, total nitrogen, total phosphorus, and pH have a greater influence on colony structure, while MP physical and chemical properties such as particle size and contact angle have less influence. Other reports (Di Pippo et al., 2020; Feng et al., 2020; Li et al., 2020) also confirmed that bacterial composition varies significantly based on marine habitat and exposure time, rather than polymer type. In the presence of heavy metals, microbial cells in biofilms can produce more extracellular polymeric substances (EPS) to protect themselves from the harsh environment (Sheng et al., 2010). EPS have been confirmed to play a very important role in biosorption of heavy metals. The biomacromolecules in EPS contain large amount of ionizable functional groups, such as hydroxyl, phosphoric, carboxyl, and amine groups, which enable EPS to absorb heavy metals. Figure 4 shows the proposed mechanisms of biofilm involved in the interactions between MPs and heavy metals. EPS can also inhibit diffusion of heavy metals in the matrix and decreases the concentration of heavy metals to sublethal concentrations. The survival of exposed microbes thus develops the ability of tolerance or resistance to heavy metals (Flemming et al., 2016). How these selection processes influence the fate of MPs and heavy metals is still unknown, and there is an urgent need to better understand these interactions (Kirstein et al., 2016; Rummel et al., 2017).

## Combined Toxic Effects and the Potential Hazards to Human Health

**FIGURE 5:**
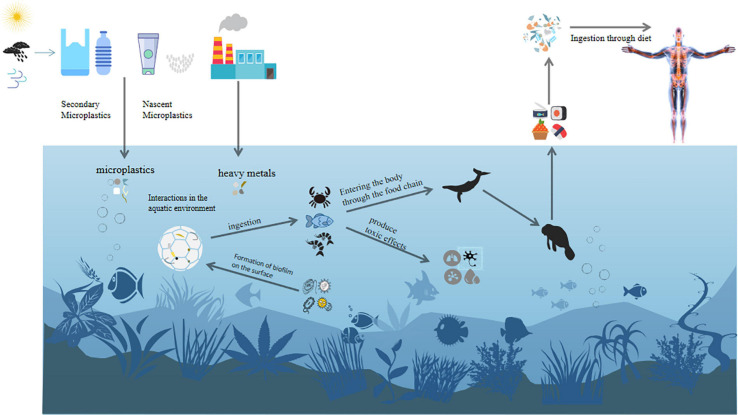
Ecological risks of MPs and heavy metals.

Contamination of aquatic systems with MPs and heavy metals is a global environmental problem of public health concern. Both MPs and heavy metals can accumulate at high level in the environment and consequently contaminate the food chains (Figure 5). The pollution of heavy metals to the environment has long been recognized (Svecevièius et al., 2014), but MPs were once regarded as relatively inert pollutants (Ashton et al., 2010). However, many studies have shown that MPs can adsorb and release heavy metals, and their combined exposure may pose a potential threat to ecological system and human being (Akhbarizadeh et al., 2017).

### Toxic Effects on Aquatic Organisms

Aquatic organisms can ingest MPs through direct ingestion, filtering ingestion, and food chain transfer, which can produce certain toxic effects when they accumulate at high levels in the body. Murphy and Quinn (2018) reported that MPs can reduce the feeding rate of freshwater snakes and cause them to produce non-lethal morphological changes, but they have no significant effect on their reproduction. Wen et al. (2018b) found that MPs can reduce the activity of acetylcholinesterase, thereby affecting neuromuscular and reducing its feeding rate. The adverse effects were more obvious when aquatic organisms were exposed to both MPs and heavy metals. Banaee et al. (2019) reported that when Cd and MPs were applied to carp, the blood biochemical and immunological indicators of carp changed significantly. The acetylcholinesterase activity in plasma and the total protein content were both reduced, and the triglycerides and cholesterol levels were elevated; thus, the immune system level was reduced, making it more susceptible to infection and death. Wen et al. (2018a) also reached the same conclusion. They found that when Cd and MPs were co-applied to Amazon discus fish, severe oxidative stress response and innate immune defense were generated compared to the administration of a single poison. However, Sun et al. (2019) showed that the combination of MPs and heavy metals can induce hippocampal oxidative damage and increase mortality, but this effect was mainly caused by heavy metals, not MPs. Yan et al. (2020) also pointed out that the adverse impacts of MPs and heavy metals on the development of marine medaka gonads was mainly due to heavy metals. Actually, those differences are understandable. With the different types of MPs and heavy metals, as well as test species, toxic effects are expected to be different. For example, cellophane is easy to accumulate in the gills, mantle, and muscles of oysters, so it has a greater impact on food intake and oxygen uptake, while polyester is more likely to accumulate in the digestive glands and has a greater impact on the absorption of protein, cholesterol, and fat (Zhu et al., 2020). As far as heavy metals are concerned, Pb can affect the nervous system by directly damaging brain cells, while Cd can selectively deposit in the kidneys and liver and cause kidney disease (Tchounwou et al., 2012). Heavy metals in different valence states show different toxicity; for example, hexavalent chromium is known to be more toxic than trivalent chromium. Furthermore, larger organisms such as sea turtles and fish are usually more resistant to MPs than microorganisms and therefore have a lower lethality rate (van Franeker et al., 2011; Kleinteich et al., 2018; Duncan et al., 2019; Oliviero et al., 2019; Wang et al., 2019b).

### Potential Hazards to Human Health

Both the World Health Organization [World Health Organization (Who), 2019] and Science Advice for Policy by European Academies (Sapea Working Group on Microplastics, 2019) stated that it is currently impossible to fully determine whether the risk of ingesting MPs exists due to the lack of human toxicity data on exposure to MPs. However, the inability to determine whether the risk exists does not mean that the risk is negligible. Therefore, many researchers have tried to use different test methods or models to assess the potential human health risks of MPs.

Food chain threat is the first thing to be considered. Robin et al. (2020) reported that polyethylene MPs were found in 15 of the 70 commercial fishes collected in Indian waters. Steer et al. (2017) also found 2.9% of the fry collected in the English Channel ingested blue fiber-based MPs. Fish were also reported to accumulate high levels of heavy metals. Tayebi and Sobhanardakani (2019) found that average levels of Cd and Pb in imported tilapia were found to be higher than the World Health Organization (WHO) maximum permissible levels (MPLs). Milatou et al. (2020) also found that total mercury levels in Mediterranean Atlantic bluefin tuna exceeded the maximum levels set by the European Commission. The presence of MPs in fish is supposed to increase their uptake of heavy metals, which will inevitably increase the health risk to humans through the food chain. There are many studies that demonstrated the wide presence of MPs and/or heavy metals in fish body (Akhbarizadeh et al., 2018; Milatou et al., 2020; Ta and Babel, 2020; Zitouni et al., 2020; Abidli et al., 2021; Covernton et al., 2021; Jonathan et al., 2021; Martinez-Tavera et al., 2021; Sarkar et al., 2021). For example, Akhbarizadeh et al. (2018) investigated MPs and metals’ concentration in muscles of both benthic and pelagic fish species from northeast of Persian Gulf and assess the risk of their consumption; Sarkar et al. (2021) detected MPs (PET and PE) and high levels of heavy metals (AS, Cd, Cr, Cu, Ni, Pb, and Zn) in both pond water and fish meat. Beside fish, MPs in other animals were also found carrying toxic metals, and positive relationships between MP ingestion and toxic metal concentration were reported (English et al., 2015; Akhbarizadeh et al., 2018). However, the reason for MP increase in heavy metals in animals is still not clear. It is reasonable to doubt the vector roles that MPs play compared to natural particles because of their relatively low abundance. In natural aquatic environments, there are many natural particulate matter that can bind heavy metals to varying degrees, including sands, clay, metal oxides and hydroxides, humus, microbes, and other small-size substances. If surface adsorption is the main sorption mechanism, the influence of other natural media should be similar or equal to that of MPs, unless heavy metal levels are higher in most plastics or animals have a preference for ingesting MPs. More investigation are needed to fully understand the mechanism of heavy metal accumulation by MPs, and how MPs compete natural particulate matter as heavy metal vectors.

A recent study found that MPs can be detected in human excreta, which confirms that MPs can indeed enter the human body through various pathways (Schwabl et al., 2019). As an effective carrier of heavy metals, MPs can form complex contaminants with heavy metals, which may affect the immune system and induce various diseases when they enter the body. The effects of heavy metals on human health have been widely reported (Zahra, 2017; Fu and Xi, 2019). For example, Hg can cause chronic neurotoxicity in humans; Pb invasion can damage the nervous system and digestive system, making people appear anemic, with low immunity, abdominal pain, and other symptoms; Cr is easy to enter human cells, causing DNA damage, and is carcinogenic and may induce genetic mutations. To more directly assess the risk to human health from the combined contamination of heavy metals and MPs, Godoy et al. (2020) used a dynamic gastrointestinal simulation device to study the bioaccessibility of Cr and Pb in MPs in the human body and found that the release rate and content of these two heavy metals were different. Cr was mainly released in the stomach, while Pb was released more in the duodenum. Liao and Yang (2020) conducted *in vitro* experiments on the whole digestive system for Cr-containing MPs and found that Cr is mainly released into the body during gastric digestion, and Cr has a higher bioavailability in degradable MPs [such as polylactic acid (PLA)] compared to non-degradable MPs (PE, PP, PVC, and PS). As an emerging persistent pollutant, MPs can be harmful to animals and human beings. The common response to MP exposure includes oxidative stress, inflammation, metabolism disruption, cytotoxicity, and translocation to other tissues (Rahman et al., 2021; Wang et al., 2021b). Due to the persistent nature of MPs, the living organisms might also get long-term exposure to ingested MPs, which can lead to chronic responses, such as necrosis, compromised immune function, and reproductive and developmental damage (Smith et al., 2018; Yin et al., 2021). The effects of chronic exposure to MPs appear to be very variable, depending on the exposure level and individual susceptibility. Furthermore, the MPs in real life are a cocktail of different MPs, non-polymers, additives, heavy metals, and pathogenic microbes. Its combined exposure is tremendously different from those of individual components, which further complicate the problem (Hirt and Body-Malapel, 2020; Rahman et al., 2021). Obviously, the risk assessment of MPs and heavy metals on human health is complex, and it is still impossible to state with certainty the extent to which the combined pollution of MPs and heavy metals is harmful to human health. Regional differences, pollution status, physiological characteristics of organisms, and dietary habits can affect the risk assessment results, so it is important to fully consider regional characteristics and applicability of the results when conducting risk assessment, which may facilitate scientific regulation. Although the risk is not clear, it is believed that the combined exposure of MPs and heavy metals can increase the adverse effects and bring unpredictable harm to biosphere. Researchers are recommended to address the knowledge gaps in understanding the toxicity of combined cumulative exposure of MPs and heavy metals and develop standardized methods for evaluating their potential risks.

### Role of Microorganisms on the Toxic Effects of MPs and Heavy Metals

MPs provide habitat for microbial communities and to some extent alter their lifestyle, metabolic pathways, and biogeochemical activities (Bryant et al., 2016). It has been shown that biofilms associated with MPs have the ability to alter nitrogen and phosphorus cycling processes in aquatic systems, and this effect is thought to be achieved through increased denitrification capacity and microbe-mediated phosphorus (P) conversion (Chen et al., 2020). Xue et al. (2020) also reported the impact of bacterial communities in plastisphere on the biogeochemical cycle, and they found that the microbes related to nitrification, denitrification, and sulfur cycles on plastic fragments have higher levels than the surrounding water. It implied that nitrogen- and sulfur-related metabolism was more vigorous in the biofilm. In addition, microorganisms can use MPs or their degradation intermediates as carbon sources (Rogers et al., 2020). Intermediate degradation products of MPs from abiotic processes, such as dissolved organic carbon (DOC) as well as methane and other hydrocarbon gasses, can also be used as potential electron donors for microbes, thus affecting the carbon cycle in aquatic systems. MPs can also play a role in the transport of trace metals. Guan et al. (2020) reported that the development of biofilms enhanced the role of MPs in the transport and fate of trace metals [Ni(II), Cu(II), Zn(II), and Cd(II)] in the aqueous environment. They found that biofilms altered the kinetics of trace metal adsorption on MPs, mainly as a result of the complexation of functional groups on the surfaces of both MPs and biofilms.

Pathogenic bacteria can travel the waters on MPs. Feng et al. (2020) found that MP surfaces are enriched with *Vibrio*, *Erythrobacteriaceae*, and *Xanthobacteriaceae*, which can cause coral bleaching and tissue damage. Sun et al. (2020) also reported that the potentially pathogenic *Vibrio* on MP surfaces can increase the ecological risk of MPs to the marine aquaculture industry. Many studies have found that the bacterial communities that accumulated on the surface of MPs were more correlated with human disease than in the water column (Wang J. et al., 2020; Xue et al., 2020). Laverty et al. (2020) isolated three human pathogens from MPs, including *Vibrio cholerae*, *Vibrio parahaemolyticus*, and *Vibrio traumatica*, which may pose a risk to aquatic ecosystems and human health. Presence of heavy metals in waters may exacerbate the risk of microbes to human health, as a study has found that heavy metals (Cu^2+^ and Zn^2+^) significantly increased the horizontal transfer of plasmids in pathogenic bacteria, which may lead to the prevalence of drug-resistant pathogenic bacteria in the water environment (Wang et al., 2021c).

Some microorganisms can become resistant to antibiotics when they are under stress. Heavy metals have been shown to increase antibiotic resistance through co-selection (Stepanauskas et al., 2006; Bednorz et al., 2013; Medardus et al., 2014). Zn, Cd, and Hg have been found to be associated with methicillin resistance on *Staphylococcus aureus* chromosomes (Ito et al., 2001; Cavaco et al., 2010). Heavy metals can persist in the natural environment for long periods of time; therefore, their contribution to the maintenance and spread of antibiotic resistance factors may be more than we expected (Baker-Austin et al., 2006; Ji et al., 2012). MPs, which act as effective carriers of both heavy metals and biofilms, thus pose a new health threat on a global scale (Imran et al., 2019).

Furthermore, microorganisms can alter the bioavailability of heavy metals by converting heavy metals into insoluble or less bioavailable valence states through redox reactions. Bacteria such as *Pseudomonas*, *Bacillus*, *Enterobacter*, and *Agrobacterium* have been shown to have high Cr (VI) reduction ability (Ohtake et al., 1987). Cr (VI) reduction can be divided into enzymatic and non-enzymatic reductions. The non-enzymatic reduction is mediated by reducing substances such as glutathione and vitamin C, whereas the enzymatic reductions are mainly catalyzed by chromate reductases, all of which are capable of reducing highly toxic Cr (VI) to Cr (III) that does not readily enter the cell. All reported chromate reductases (e.g., ChrR, YieF, NemA, and LpDH) use flavin as a cofactor and NAD(P)H as an electron donor, and the electron transfer mechanisms are different (Thatoi et al., 2014; Xia et al., 2021). Soluble reductase (SR) and membrane-bound reductase (MR) can also be used as Cr (VI) reductases, and the electron donors utilized are mainly NADH or endogenous electron reserves. From this point of view, since MPs can act as carriers of both heavy metals and microorganisms, the biofilms formed on their surfaces seem to alter the bioavailability of heavy metals more efficiently, thus exerting an unneglectable impact on the toxic effect of heavy metals.

## Conclusion and Outlook

Evidence has shown the ubiquitous presence of MPs and heavy metals in the water environment. This paper give a perspective review on the interactions between MPs and heavy metals. The result of bibliometric analysis showed that contamination, mechanism, sediment, MPs, and pollution are the burst keywords for the latest 3 years, indicating that MP pollution is receiving widespread attention. Keyword co-occurrence mapping analysis indicated that biofilm has a high betweenness centrality. The environmental factors, including microbes, exert important influence on the interactions between MPs and heavy metals, and the combined toxic effects and the potential hazards to human health merit further concern. Our result demonstrated that microorganisms play an important role in the interaction network between heavy metals and MPs in the water environment; however, their combined effects on the aquatic ecosystem and human health are largely unknown.

Although a tremendous amount of work has been conducted in this field, substantial data gaps exist. To fill these knowledge gaps, the following issues deserve further attention:

(1) The adsorption capacity of heavy metals by MPs under multiple environmental factors is needed to evaluate the influence of environmental factors on the interactions, especially the influence of biofilm. Many of the experimental data were obtained under controlled laboratory conditions, while the adsorption of heavy metals by MPs in the natural environment is complex.
(2) The ecological and toxic effects of MPs at environmentally realistic concentrations are needed. The toxicity tests generally use new and pristine MPs with defined size and expose the organisms to high MP concentrations. Furthermore, MPs can be degraded by the biotic/abiotic factors; however, the concentration and toxicity of these smaller MPs (nanoscale and even smaller) are largely unknown, which makes risk assessment difficult.
(3) Although there are many studies on MPs and heavy metals, there is still a lack of detailed explanation on the role of microbes on their interactions. There is an urgent need for the comprehensive methods for the rapid and accurate sampling, characterization, analysis, and evaluation of the composite pollutants and their combined risks.
(4) There are still no water quality criteria for MP-related pollutants for the control of MP emissions, protection of human health, and ecosystem safety. Therefore, the cooperative efforts of scientists, policy makers, government officials, general public, and the international communities are urgently needed in the future.

## Author Contributions

SL: investigation, data curation, and writing—original draft. JW, YD, and HL: resources, investigation, and data curation. JL: resources, investigation, and data curation. PZ: resources, investigation, and data curation. XC: methodology and data curation. ZW: conceptualization and writing—review and editing. XL: conceptualization, supervision, and writing—review and editing. All authors contributed to the article and approved the submitted version.

## Conflict of Interest

The authors declare that the research was conducted in the absence of any commercial or financial relationships that could be construed as a potential conflict of interest.
